# Optimizing the role of impact ionization in conventional insulators

**DOI:** 10.1038/s41598-019-56974-y

**Published:** 2019-12-31

**Authors:** Efstratios Manousakis

**Affiliations:** 1 Department of Physics and National High Magnetic Field Laboratory, Florida State University, Tallahassee, FL 32306-4350 USA; 2 Department of Physics, University of Athens, Panepistimioupolis, Zografos 157 84 Athens, Greece

**Keywords:** Electronic structure, Electronic properties and materials

## Abstract

A mechanism for multiple carrier generation through impact ionization (IA) proposed earlier for bulk systems of strongly correlated insulators is generalized to the case of conventional insulators that contain localized bands a few eV above and below the highest occupied band. Specifically, we study the case of hybridization of localized orbitals with more dispersive bands near the Fermi level, where the generated multiple carriers, which ultimately decay to the edges of the dispersive bands by means of IA processes, acquire lighter mass and this could allow their more efficient separation before recombination. We argue that this may be applicable to the case of halide perovskites and it could be one of the reasons for their observed photovoltaic efficiency. We discuss the criteria one should use to uncover the appropriate material in order to harvest the optimum effect of IA for the spectrum of the solar photon energy distribution.

## Introduction

Most of the traditional photo-active semiconductors can produce at most one electron-hole pair when absorbing a single photon, which imposes a limit on the energy conversion efficiency of solar cells^1^. Theoretically, the quantum yield and the energy efficiency of various types of optoelectronic devices can be significantly increased by means of multi-carrier generation (MCG) through the process of impact ionization(IA). IA in conventional semiconductors is typically negligible because of weak interactions between excited charge carriers. However, the mechanism of IA was thought to be applicable in non-bulk finite geometries, such as quantum dots or nanocrystals, because the spatial confinement lifts the required momentum matching during carrier transitions and prevents the generation of truly long-wavelength phonons. As a result IA was observed in nanocrystals^2^, and a considerable IA rate was discovered in some semiconductor quantum dots^3–5^. Unfortunately, the IA in nanocrystals is not very strong and quantum dots have their inherent limitations in charge transport, which has inhibited their usefulness in optoelectronic devices.

Several years ago, we pointed out^6^ that high IA rates can be realized in the bulk of properly selected materials, and, specifically, a narrow-gap Mott insulator can be a good candidate. This idea implies that some oxide perovskite materials maybe good candidates for MCG. The proposal was later extended to the more general family of strongly correlated insulators^7^ (SCIs) where localized flat bands exist near the Fermi level. Subsequent theoretical^8–12^ and experimental^13–26^ investigations on oxide perovskite and related materials have shed light on important aspects of this initial theoretical proposal. In particular a pump-probe optical study^16^ of the low-temperature phase of VO_2_, a prototypical Mott insulator, shows evidence of IA. This study, however, was plagued by the fact that a photo-induced metal to insulator transition occurs in VO_2_ when illuminated.

Realizing an SCI in materials which at the same time fulfill the requirements for an efficient photo-voltaic (PV) cell may be a difficult task. After our theoretical proposal^6^ a different family of perovskites, halide perovskites^27,28^, which are not believed to be SCIs, shows a relatively good PV efficiency. In this paper we show that this requirement for a material to be an SCI in order to have high IA rate can be further generalized to include a very general class of materials which are conventional band insulators with localized bands near the Fermi level, which may hybridize with dispersive bands. Namely, it can be applied to an insulating material where its highest occupied band and its lowest empty band have relatively flat regions because they hybridize with localized atomic orbitals. Our definition of a “flat band” is that the bandwidth is small or comparable to the strength of the Coulomb-repulsion energy-scale which is felt by a pair of electrons which occupies the atomic orbitals forming these bands. Electrons or holes of flat bands, however, are “heavier” than those in dispersive bands because they are more localized as compared to those in dispersive bands. Therefore, it is desirable that another more dispersive band be present within a few eV above and/or below the Fermi level which strongly hybridize with the localized atomic orbitals. This hybridization can act effectively, because after the impact ionization process takes place, the created electron-hole pairs can decay into the more dispersive parts of the band and move with a smaller mass. This transformation of the multiple electron/hole carriers to lighter mass carriers allows their more effective separation.

We argue that it is possible some halide perovskite materials^29^ (or possibly the well-known methylammonium or formamidinium lead halides^30–32^) may already fulfill these criteria and, therefore, their relatively high efficiency could be, in part, due to MCG in these materials. However, their efficiency could be improved further by utilizing the conclusions presented in this work. Since the practical use of the halide perovskite materials is seriously hindered by the fact that they are unstable, the essence of the ideas and criteria presented in this paper are very simple and can be applied to the quest for a variety of other materials where IA may be effective.

In the following section we present the idea and the formalism/method involved in order to calculate IA rates in materials. In the same section, we also present our results for carefully selected models in order to clearly illustrate the factors that play a key role in an on-going search for real materials. General conclusions drawn from this work are presented in the Discussion section.

## Model and Results

### Formulation of the general idea

The IA mechanism discussed in ref. ^6^ for Mott insulators and in ref. ^7^ more generally for SCIs, utilizes the presence of localized bands near the Fermi level. In the former case, even the origin of the gap, which is necessary for realizing the PV effect, is the result of strong electron correlations. In the latter case, the concept was applied to materials which are not necessarily Mott insulators but the presence of localized orbitals near the Fermi level leads to strong electron correlations. For example, in VO_2_, where the gap opens because of a Peierls instability^33–35^, the calculated IA rate was found to be higher than ${10}^{14}/sec$ in the region of the solar radiation. It was further demonstrated that this enhancement was due to the contribution of the vanadium *d* orbitals.

While the effect of strong electron correlations is needed in order to produce high IA, usually as a result of these correlations the effective masses for the electron and holes become large and this makes the separation of the created multi-electron/hole pairs more difficult. In the present work, we consider the case when such localized orbitals hybridize with other more dispersive bands in the same energy range. We demonstrate that this class of materials can provide a better chance to solve the problem of carrier separation by endowing the carriers with a low mass without lowering the IA rates below the threshold needed in order to prevent other dissipation processes to act to “cool” the carriers.

In order to discuss the idea we will write down a generic Hamiltonian of the form:

1 $$\hat{H}=\sum _{{\bf{k}},\nu ,\sigma }\,{\varepsilon }_{{\bf{k}}\nu \sigma }^{(\pm )}{c}_{{\bf{k}}\nu \sigma }^{(\pm )\dagger }{c}_{{\bf{k}}\nu \sigma }^{(\pm )}+\hat{U},\,\hat{U}=\sum _{m}\,{U}_{m}\sum _{{\bf{R}}}\,{d}_{m{\bf{R}}\uparrow }^{\dagger }{d}_{m{\bf{R}}\uparrow }{d}_{m{\bf{R}}\downarrow }^{\dagger }{d}_{m{\bf{R}}\downarrow },$$

where the last term represents the Hubbard on-size Coulomb repulsion ${U}_{m}$ when pairs of electrons occupy the same localized orbital $M$. We have ignored the spin-orbit coupling in this formalism for simplicity. The first term denotes the conduction (+) and the valence (−) bands in which the creation operators create Bloch states out of the following atomic orbitals:

2 $${c}_{{\bf{k}}\nu \sigma }^{(\pm )\dagger }=\frac{1}{\sqrt{N}}\sum _{{\bf{R}}}\,{e}^{i{\bf{k}}\cdot {\bf{R}}}{c}_{{\bf{R}}\nu \sigma }^{(\pm )\dagger },$$

3 $${c}_{{\bf{R}}\nu \sigma }^{(\pm )\dagger }=\frac{1}{\sqrt{N}}[\sum _{n}\,{\alpha }_{\nu n{\bf{k}}\sigma }^{(\pm )}{p}_{n{\bf{R}}\sigma }^{\dagger }+\sum _{m}\,{\beta }_{\nu m{\bf{k}}\sigma }^{(\pm )}{d}_{m{\bf{R}}\sigma }^{\dagger }],$$

where $N$ is the number of unit cells in a periodic system. The operators ${p}_{n{\bf{R}}\sigma }^{\dagger }$ and ${d}_{m{\bf{R}}\sigma }^{\dagger }$ create states $|{p}_{n}{\bf{R}}\rangle |\sigma \rangle$ and $|{d}_{m}{\bf{R}}\rangle |\sigma \rangle$, where $p$ and $d$ denote orbitals in the unit cell specified by the lattice vector ${\bf{R}}$ and the spin state $|\sigma \rangle$. In the absence of spin-orbit coupling, which has been neglected for simplicity, the bands and orbitals are characterized by $\sigma$. Notice that without loss of generality we have assumed that these bands are of only $p$ and $d$ character, however, they also have some $s$ character too. We will assume that the coefficients ${\alpha }^{(\pm )}$ and ${\beta }^{(\pm )}$ above are non-zero for all bands. We use $d$ as the prototypical orbital which tends to form more localized states. However, localization can also occur because of a small hopping amplitude due to physical separation between atoms in the crystal. This will also increase the on-site Coulomb repulsion between two electrons that occupy such an orbital, purely because of the fact that the electronic wavefunction is localized and does not spread too much in space.

**Figure 1:**
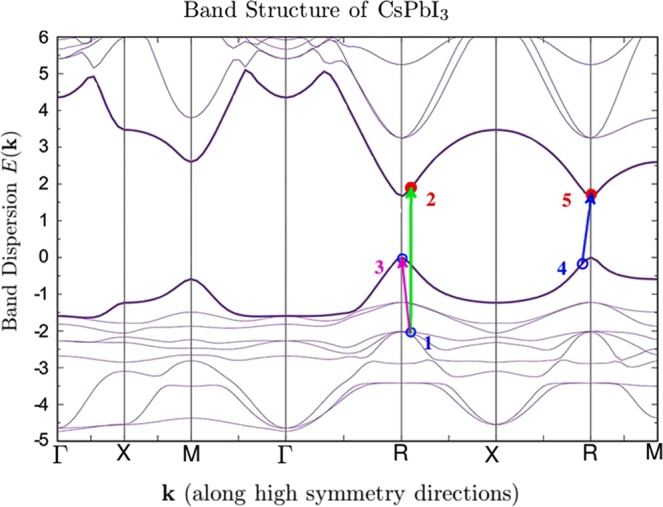
The bands of CsPbI_3_ taken from the calculation of ref. ^36^. We have added an example of electron/hole photo-excitation where the incident photon promotes an electron from the occupied state 1 to the empty state 2. Within a time scale shorter than ${10}^{-14}$ seconds, the created hole at state 1 decays into the state 3 near the top of the valence band and at the same time the on-site Coulomb repulsion promotes the electron occupying the valence state 4 to the empty state 5 near the conduction band edge. This process causes the incident solar photon to produce two electrons and two holes as a final product.

The mechanism discussed in this paper could be applicable to the well-known methylammonium lead halides^30–32^ as they exhibit some features (i.e., combination of flat and dispersive bands near the highest occupied band) which can lead to a high IA rate. The macroscopically long diffusion lengths observed in such materials^31^ could also be in part a possible consequence of high IA rates. However, in order to be definitive about the origin of their relative high efficiency, optical pump-and-probe experiments, such as those carried out in ref. ^16^ for VO_2_, are needed. Let us consider the case of the halide perovskites CsPbCl_3_, and CsPbI_3_. The reason for considering these materials is that MCG has been observed in CsPbI_3_^29^, and the band-structure is available for these caesium lead halide perovskites^36^ (See extended data Fig. 1 of this citation). Notice that in the case of CsPbI_3_ the highest occupied valence band and some other bands just below it are flat around the $\Gamma$, $M$ and *X* high symmetry points. Notice that they become very dispersive near the topmost point of the valence band where the photo-created holes will land after they lose their excess energy due to IA or other processes such as phonon-emission. This is a good example of what we are discussing in the present paper, namely a mixture of a localized orbital, such as $d$ with a more dispersive band due to an $s$ or $p$ orbital. Notice that if a solar photon is absorbed by this material there are many bands with significant $d$ content which can host the photo-created hole. These bands are generally flat as may be seen in Fig. 1. Within a few eV above and below the highest occupied state, there are bands with very dispersive conduction-band bottoms or valence-band tops which are generally of $s$ and $p$ character with some $d$ content.

**Figure 2:**
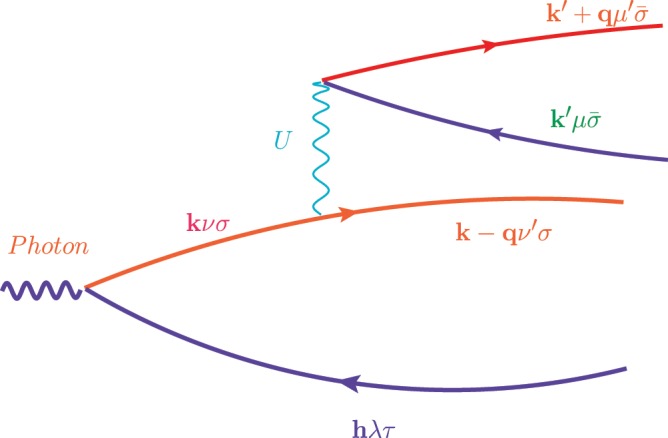
The decay of the photo-excited electron into a state of two-electrons and one-hole from the bands discussed in the text. A very similar process exists where the photo-created hole (blue line) decays into a state involving two-holes and one conduction electron. The latter is the process discussed in Fig. 1.

Now, let us consider the impact ionization rate of a photo-created electron-hole pair from a valence band to a conduction band which consists of a significant contribution of $d$ orbitals, as seems to be the case for CsPbI_3_. Namely, there are states just below the highest occupied state which are obtained as a hybridization of $d$ and $p$ orbitals and produce bands which have a combination of flat and dispersive parts as we travel the BZ. The created electron or hole or both, when is in the band labeled by n with momentum ${\bf{k}}$, under properly chosen conditions and systems to be discussed below, has a significant probability to decay into the lower energy conduction or valence band by transferring part of its energy and momentum to another electron-hole pair as depicted in Fig. 2. The order of magnitude of the impact ionization rate is given as

4 $${\Gamma }_{{\bf{k}}\nu }=\frac{2\pi }{\hslash }\sum _{\nu ^{\prime} \mu \mu ^{\prime} \sigma }\,\sum _{{\bf{q}},{\bf{k}}{\boldsymbol{^{\prime} }}}\,|{M}_{{\bf{k}},{\bf{k}}{\boldsymbol{^{\prime} }},{\bf{q}}}^{\nu \nu ^{\prime} \mu \mu ^{\prime} \sigma }{|}^{2}\delta ({\omega }_{{\bf{k}},{\bf{k}}{\boldsymbol{^{\prime} }},{\bf{q}}}^{\nu \nu ^{\prime} \mu \mu ^{\prime} \sigma }),$$

5 $${M}_{{\bf{k}},{\bf{k}}{\boldsymbol{^{\prime} }},{\bf{q}}}^{\nu \nu ^{\prime} \mu \mu ^{\prime} \sigma }\equiv \langle {c}_{{\bf{k}}\nu \sigma }^{(+)}\hat{U}{c}_{{\bf{k}}-{\bf{q}}\nu ^{\prime} \sigma }^{(+)\dagger }{c}_{{\bf{k}}{\boldsymbol{^{\prime} }}\mu \bar{\sigma }}^{(-)}{c}_{{\bf{k}}{\boldsymbol{^{\prime} }}+{\bf{q}}\mu ^{\prime} \bar{\sigma }}^{(+)\dagger }\rangle ,$$

6 $${\omega }_{{\bf{k}},{\bf{k}}{\boldsymbol{^{\prime} }},{\bf{q}}}^{\nu \nu ^{\prime} \mu \mu ^{\prime} \sigma }\equiv {\varepsilon }_{{\bf{k}}\nu \sigma }^{(+)}-{\varepsilon }_{{\bf{k}}-{\bf{q}}\nu ^{\prime} \sigma }^{(+)}+{\varepsilon }_{{\bf{k}}{\boldsymbol{^{\prime} }}\mu \bar{\sigma }}^{(-)}-{\varepsilon }_{{\bf{k}}{\boldsymbol{^{\prime} }}+{\bf{q}}\mu ^{\prime} \bar{\sigma }}^{(+)},$$

where $\sigma$ takes the two values of the spin and $\bar{\sigma }$ is the opposite spin eigenvalue. The expectation value above is taken with respect to the ground state in which all single particle states up to the highest occupied band are filled. Using the Hubbard term given by Eq. 1 we can calculate the matrix element required in the above expression:

7 $${M}_{{\bf{k}},{\bf{k}}{\boldsymbol{^{\prime} }},{\bf{q}}}^{\nu \nu ^{\prime} \mu \mu ^{\prime} \sigma }=\frac{1}{N}\sum _{m}\,{U}_{m}{\beta }_{\nu m{\bf{k}}\sigma }^{(+)}{[{\beta }_{\nu ^{\prime} m{\bf{k}}-{\bf{q}}\sigma }^{(+)}]}^{\ast }{\beta }_{\mu m{\bf{k}}{\boldsymbol{^{\prime} }}=\bar{\sigma }}^{(-)}{[{\beta }_{\mu ^{\prime} m{\bf{k}}{\boldsymbol{^{\prime} }}+{\bf{q}}\bar{\sigma }}^{(+)}]}^{\ast },$$

Where $|\bar{\sigma }\rangle$ stands for the orthogonal state to the state $|\sigma \rangle$. The above expression is very sensitive to the mixture coefficients entering in Eq. 7 for the matrix element and to the values of $U$. In general one can expect this to be significantly higher than 10^13^ sec^−1^ which is faster than phonon relaxation time scales. The question we wish to address in this paper is, by inspecting this expression, what can we say about the parameter values which optimize the IA rate for energy values of ${\varepsilon }_{{\bf{k}}\nu \sigma }$ in the solar spectrum. First, we consider simple examples, next, in order to further clarify the idea.

### Demonstration with simple models

We consider the hybridization of a localized orbital, such as an $f$ or a $d$ orbital, with a dispersive band which leads to an opening of a gap

8 $$\begin{array}{rcl}{\hat{H}}_{TB} & = & {\varepsilon }_{d}\sum _{{\bf{k}},\sigma }\,{d}_{{\bf{k}}\sigma }^{\dagger }{d}_{{\bf{k}}\sigma }+\sum _{{\bf{k}}\sigma }\,{\varepsilon }_{{\bf{k}}}^{p}{p}_{\underline{{\bf{k}}}\sigma }^{\dagger }{p}_{{\bf{k}}\sigma }\\  &  & -\sum _{{\bf{k}}\sigma }\,{V}_{pd}({\bf{k}})({p}_{{\bf{k}}\sigma }^{\dagger }{d}_{{\bf{k}}\sigma }+h.c\mathrm{)}.\end{array}$$

The diagonalization leads to the following two simple bands

9 $${\hat{H}}_{TB}=\sum _{{\bf{k}}\sigma }\,{\varepsilon }_{{\bf{k}}}^{(+)}{c}_{{\bf{k}}\sigma }^{(+)\dagger }{c}_{{\bf{k}}\sigma }^{(+)}+\sum _{{\bf{k}},\sigma }{\varepsilon }_{{\bf{k}}\sigma }^{(-)}{c}_{{\bf{k}}\sigma }^{(-)\dagger }{c}_{{\bf{k}}\sigma }^{(-)},$$

10 $${\varepsilon }_{{\bf{k}}}^{(\pm )}={\varepsilon }_{{\bf{k}}}^{+}\pm \Delta ({\bf{k}}),$$

11 $${\varepsilon }_{{\bf{k}}}^{\pm }\equiv \frac{{\varepsilon }_{{\bf{k}}}^{(p)}\pm {\varepsilon }_{d}}{2}\,\Delta ({\bf{k}})\equiv \sqrt{{({\varepsilon }_{{\bf{k}}}^{-})}^{2}+{V}_{pd}{({\bf{k}})}^{2}}.$$

The operators ${c}_{{\bf{k}}\sigma }^{(\pm )}$ are given by the expression Eq. 3 without the band index, because we are dealing with only one conduction and one valence band in the present example, where the coefficients in the expression are given as follows:

12 $${\alpha }_{{\bf{k}}\sigma }^{(\pm )}=\frac{1}{\sqrt{2}}\frac{1}{{\mathrm{(1}+{\lambda }_{{\bf{k}}\sigma }^{2})}^{\mathrm{1/4}}}\frac{1}{\sqrt{\sqrt{1+{\lambda }_{{\bf{k}}\sigma }^{2}}\mp {\lambda }_{{\bf{k}}\sigma }}},$$

13 $${\beta }_{{\bf{k}}\sigma }^{(\pm )}=({\lambda }_{{\bf{k}}\sigma }\mp \sqrt{1+{\lambda }_{{\bf{k}}\sigma }^{2}}){\alpha }_{{\bf{k}}\sigma }^{(\pm )},\,{\lambda }_{{\bf{k}}\sigma }={\varepsilon }_{{\bf{k}}}^{-}/{V}_{pd}({\bf{k}}\mathrm{)}.$$

Let us work in two-dimensions and take for the dispersive $p$-band

14 $${e}_{{\bf{k}}}^{(p)}=-2t(cos({k}_{x}a)+cos({k}_{y}a))$$

which is the dispersion of a square lattice tight-binding (TB) model with nearest hopping $t$. We will consider four different cases for the hybridization function ${V}_{pd}({\bf{k}})$.

**Figure 3:**
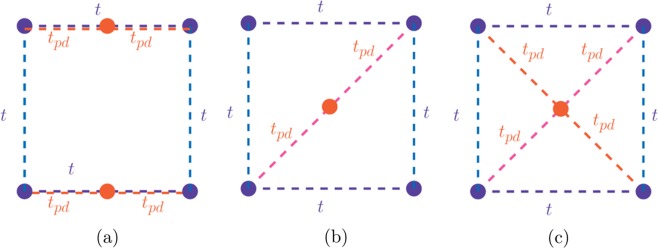
Unit cells which lead to different ${\bf{k}}$-dependence in the hybridization matrix elements. The “blue” (“red”) atom represents the atom which carriers the $p$-like ($d$-like, localized) orbital. There is a non-zero hopping parameter $t$ from one “blue” atom to the other neighboring “blue” atoms and a non-zero hopping ${t}_{pd}$ from a “blue” atom to a “red” atom. (**a**) The localized atom is in the middle of line connecting the two “blue” atoms along the $x$-direction. (**b**) The localized atom is in the unit cell center and there is only hopping (${t}_{pd}$) along one of the diagonal directions as shown. (**c**) The localized atom is in the unit cell center and there is hopping (${t}_{pd}$) along both of the diagonal directions as shown.
- The case where ${V}_{pd}({\bf{k}})=-\,2{t}_{pd}\,\cos \,(({k}_{x}+{k}_{y})a\mathrm{/2)}$ which corresponds to the square lattice with a unit cell shown in Fig. 3(b).
- The case where ${V}_{pd}({\bf{k}})=-\,4{t}_{pd}\,\cos \,({k}_{x}a\mathrm{/2)}cos({k}_{y}a\mathrm{/2)}$, which corresponds to the square lattice with a unit cell shown in Fig. 3(c).
- The case where ${V}_{pd}({\bf{k}})$ is constant independent of ${\bf{k}}$.

- The case where ${V}_{pd}({\bf{k}})=-\,2{t}_{pd}\,\cos \,({k}_{x}a\mathrm{/2)}$ which corresponds to the square lattice with a unit cell shown in Fig. 3(a).

**Figure 4:**
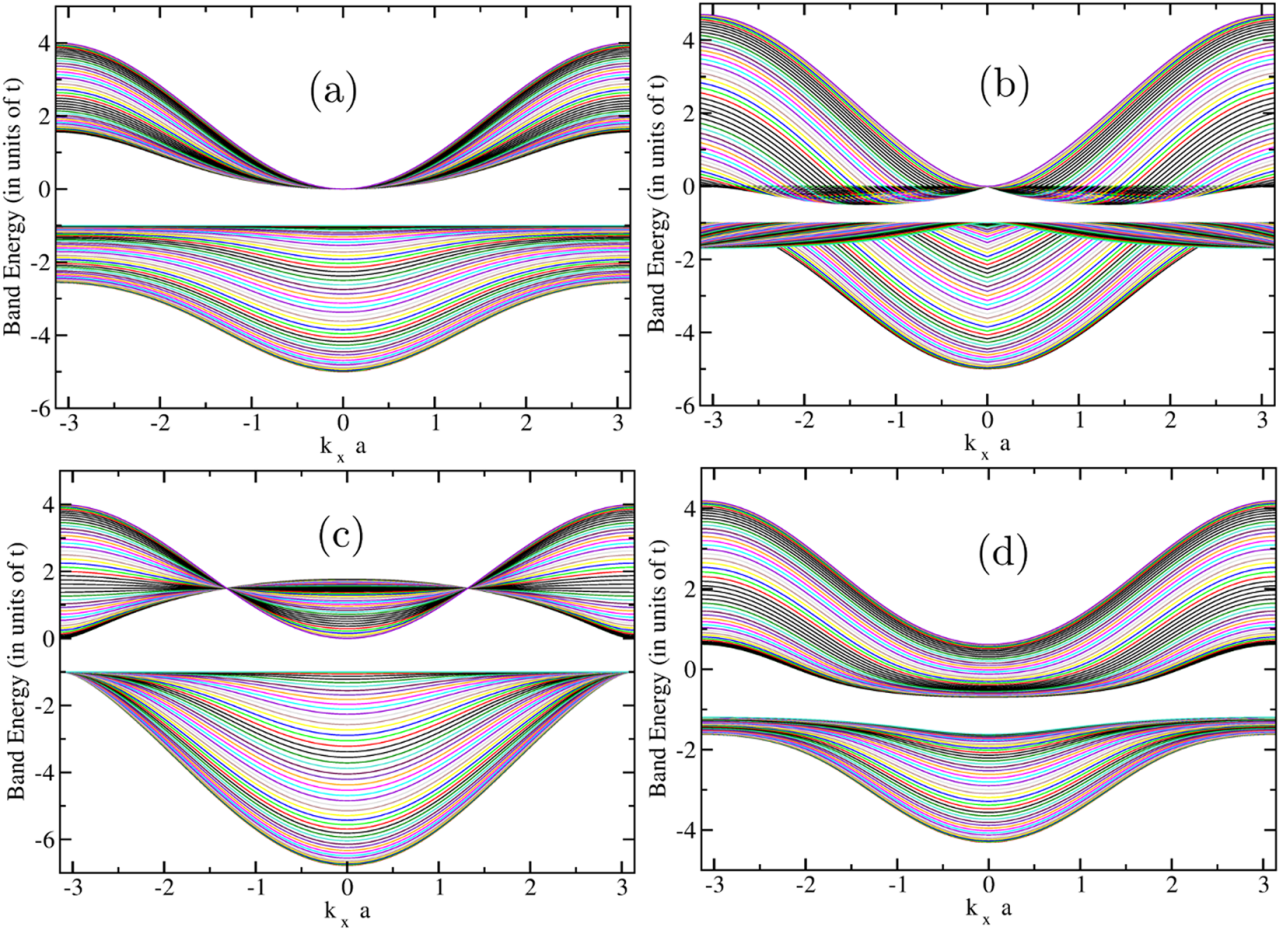
The bands of the hybridization Hamiltonian given by Eq. 8 of our example, shown as ribbons when plotting them vs ${k}_{x}$ for all values of ${k}_{y}$. The panels (a–d) correspond to the four cases of different hybridization discussed in the text.

For simplicity, in all cases we take ${\varepsilon }_{d}=-\,1$. The bands for the cases (a), (b), (c), and (d) are respectively shown in Fig. 4(a–d) where the ribbons represent the various bands obtained when plotting ${\varepsilon }_{({k}_{x},{k}_{y})}^{(\pm )}$ vs ${k}_{x}$ for all values of ${k}_{y}$. While the BZ is smaller than the range of ${k}_{x}$ and ${k}_{y}$ shown in the Figure, we have used a larger range for simplicity. Notice that the bands have a combination of dispersive and flat landscape because of the mixing of the localized band with the dispersive band.

**Figure 5:**
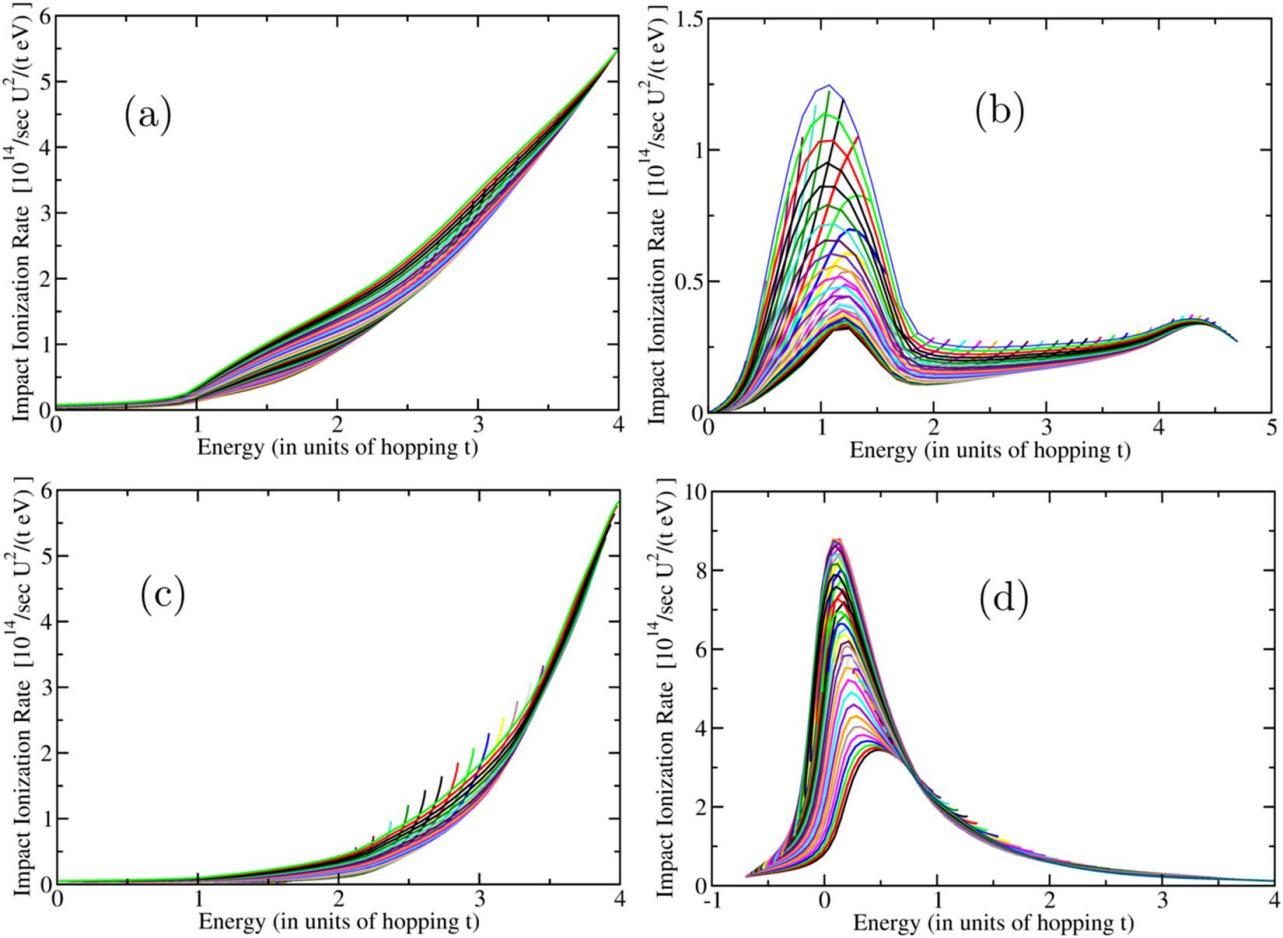
The calculated IA rate for the example model considered in the present work. It is given in units of ${10}^{14}/sec{U}^{2}/(eVt)$. Just like in the case of the ribbons presented in Fig. 4, each line is plotted for all values of ${k}_{y}$ in the BZ for a fixed value of ${k}_{x}$. The finite-size k-point mesh yields discrete lines as opposed to ribbons of accessible values for the IA rate. The panels (a–d) correspond to the four cases of different hybridization discussed in the text.

Treating the Hubbard term as in Eq. 1 within the mean-field factorization we calculate the bands in the presence of the $U$-term. Such a term adds only an overall constant to the energy and it does not change the computation of the impact ionization rate resulting from the residual Hubbard interaction. Next, we computed the impact-ionization rate using the Eq. 6 where the matrix element given by Eq. 7 is calculated using the coefficients of the Bloch eigenstates of the TB Hamiltonian. The results for the IA rate are shown in Fig. 5 for the all the values of ${\bf{k}}$ in the BZ.

Notice that we can choose the hopping matrix element $t$ and the hybridization parameter (${t}_{pd}$) in such a way so that the bands fall inside the solar spectrum. When we do that (take for example $t=1$ eV) we notice that the IA rate is very high (significantly greater than ${10}^{15}/sec$) for very modest values of $U/t$ of order 3. Therefore, this result is very promising when we need to deal with many bands (see discussion in the next Section) where the projections of the Bloch states to the localized orbitals is fragmented and can become small.

## Discussion

By having a localized $d$ orbital mixed with other dispersive bands near the Fermi level, we have been able to achieve two requirements for better PV efficiency through the IA process:

1. Conversion of the energy of the solar photon into more than just a single electron/hole pair via IA. The rates of IA shown in Fig. 5 for our example are higher than ${(U/{\rm{eV}})}^{2}{10}^{14}se{c}^{-1}$ and this rate is much higher than phonon (or other known) dissipation processes.
2. The electron/hole bands closer to the Fermi level have dispersive regions near their bottom/top where the electron/hole carriers ultimately land, which can endow the carriers with light-mass as compared to the case of SCIs. This will allow fast carrier separation and extraction before recombination can take place.

There are many classes of insulating materials containing transition metals (TM) which can be investigated, such as, using a TM at the octahedra center in halide perovskites as was done in ref. ^29^, or in oxide perovskites, using the criteria discussed here to examine their band-structure and the projected density of states a few eV above and below the highest occupied bands. While the PV efficiency of a material depends on many other factors, next, we would like to focus and discuss only general criteria in order to optimize the IA ionization process in such a way to also produce low-mass carriers to make possible their extraction before recombination.

First, the combination of a localized and a dispersive band increases the bandwidth $W$ which should decrease the IA rate $\Gamma$ as it depends on it as $\mathrm{1/}W$. The expression for $\Gamma$ depends on the joint density of states ${\cal{D}}$, or roughly on $\mathrm{1/}W$; therefore, having flat bands (i.e., a significant contribution of localized orbitals in these bands) inside the energy window of the solar radiation above and below the Fermi energy is important because they will enhance ${\cal{D}}$. However, because the dependence of the IA on the value of $U$, associated with the localized $d$ orbitals which participate in the formation of both the conduction and valence bands, is quadratic we can icrease the coefficient of the localized orbital contributing to the bands and to choose more atoms with more localized atomic orbitals which increase the value of $U$ and compensate for the effect of the larger $W$.

Second, we notice that the expression Eq. 7 for the matrix element $M$ contains the squares of the amplitude of the localized orbitals in the bands involved in the IA process. At first, one may be inclined to look for pure localized states in this energy window above the Fermi energy. However, this would result in totally flat bands and, thus, the photo-generated carriers would have large mass and this hinders the separation of the electrons from the holes in a short period of time to minimize carrier recombination. For this reason it might be desirable to have a mixture of localized orbitals with some $p$ orbitals. The presence of many orbitals within a unit cell will cause a distribution of the weights ${\beta }^{(\pm )}$ in the expression of the rate $\Gamma$ and since they are all less than unity, they reduce the factor $M$. The largest value of this factor is achieved with just one localized and one $p$ orbital contributing with equal weight to all four bands which will be produced by the two orbitals and their bonding (valence bands) and antiboding (conduction bands) combinations. Decimating the weights by including many different orbitals in the construction of such a band with about the same weights, reduces the IA rate. It is therefore, desirable to include the minimum possible number of orbitals in order to maximize the matrix element $M$.

Last, we also need to keep in mind that there is no reason to increase the IA rate more than the threshold value required, which is the rate of the other dissipation processes inherent in the material. As long as $\Gamma$ is greater than this theshold rate, ultimately, the photo-excited carriers will relax by creating additional electron-hole pairs via this process. Therefore, reducing the value of $M$ by allowing the localized orbital to hybridize with more dispersive bands may not introduce a significant issue in converting the solar energy into multiple carrier excitations, as long as $\Gamma$ remains larger than the lowest cutoff.

## References

[1] Shockley W, Queisser HJ (1961). Detailed balance limit of efficiency of p-n junction solar cells. J. Appl. Phys..

[2] Semonin OE (2011). Peak external photocurrent quantum efficiency exceeding 100% via meg in a quantum dot solar cell. Science.

[3] Nozik AJ (2008). Multiple exciton generation in semiconductor quantum dots. Chem. Phys. Lett..

[4] Nozik Arthur J (2001). SPECTROSCOPY ANDHOTELECTRONRELAXATIONDYNAMICS INSEMICONDUCTORQUANTUMWELLS ANDQUANTUMDOTS. Annual Review of Physical Chemistry.

[5] Hanna MC, Beard MC, Nozik AJ (2012). Effect of solar concentration on the thermodynamic power conversion efficiency of quantum-dot solar cells exhibiting multiple exciton generation. The J. Phys. Chem. Lett..

[6] Manousakis E (2010). Photovoltaic effect for narrow-gap mott insulators. Phys. Rev. B.

[7] Coulter JE, Manousakis E, Gali A (2014). Optoelectronic excitations and photovoltaic effect in strongly correlated materials. Phys. Rev. B.

[8] Werner P, Held K, Eckstein M (2014). Role of impact ionization in the thermalization of photoexcited mott insulators. Phys. Rev. B.

[9] Charlebois M, Hassan SR, Karan R, Sénéchal D, Tremblay A-MS (2013). Mott *p*-*n* junctions in layered materials. Phys. Rev. B.

[10] Sorantin ME, Dorda A, Held K, Arrigoni E (2018). Impact ionization processes in the steady state of a driven mottinsulating layer coupled to metallic leads. Phys. Rev. B.

[11] Wais M (2018). Quantum boltzmann equation for strongly correlated systems: Comparison to dynamical mean field theory. Phys. Rev. B.

[12] Petocchi, F., Beck, S., Ederer, C. & Werner, P. Hund excitations and the efficiency of Mott solar cells. *Phys. Rev. B.* **100**, 075147, 10.1103/PhysRevB.100.075147 (2019).

[13] Wang, L. *et al*. Device performance of the mott insulator lavo3 as a photovoltaic material. *Phys. Rev. Appl*. 3, 064015, 10.1103/PhysRevApplied.3.064015 (2015).

[14] Zhou Y, Ramanathan S (2013). Gan/vo2 heteroepitaxial p-n junctions: Band offset and minority carrier dynamics. J. Appl. Phys..

[15] Wang X, Gao H (2015). Distinguishing the photothermal and photoinjection effects in vanadium dioxide nanowires. Nano Lett..

[16] Holleman J (2016). Evidence for impact ionization in vanadium dioxide. Phys. Rev. B.

[17] Zhang H-T (2017). High-quality lavo3 films as solar energy conversion material. ACS Appl. Mater. & Interfaces.

[18] Liu H-J (2018). Giant photoresponse in quantized srruo3 monolayer at oxide interfaces. ACS Photonics.

[19] Ahmadi-Majlan K (2018). Tuning metal-insulator behavior in latio3/srtio3 heterostructures integrated directly on si(100) through control of atomic layer thickness. Appl. Phys. Lett..

[20] Jellite M (2018). Investigation of lavo3 based compounds as a photovoltaic absorber. Sol. Energy.

[21] Kim C, Park H, Marianetti CA (2015). New class of planar ferroelectric mott insulators via first-principles design. Phys. Rev. B.

[22] Sabou, F. C., Bodington, N. & Marston, J. B. Rectification by doped mott-insulator junctions. In *2012 Lester Eastman Conference on High Performance Devices (LEC)*, 1–4, 10.1109/lec.2012.6410983 (2012).

[23] Kumar, A. & k. Pandey, S. Design and performance analysis of perovskite solar cell. In *2018 International Conference on Numerical Simulation of Optoelectronic Devices (NUSOD)*, 69–70, 10.1109/NUSOD.2018.8570235 (2018).

[24] Fix Thomas (2019). Oxide and Ferroelectric Solar Cells. Advanced Micro- and Nanomaterials for Photovoltaics.

[25] El-Mellouhi F (2019). Enhancing the electronic dimensionality of hybrid organic–inorganic frameworks by hydrogen bonded molecular cations. Mater. Horiz..

[26] Celindano C (2019). Probing the growth window of lavo3 perovskites thin films elaborated using magnetron co-sputtering. Ceram. Int..

[27] Chen P-Y (2014). Environmentally responsible fabrication of efficient perovskite solar cells from recycled car batteries. Energy Environ. Sci..

[28] Manser JS, Christians JA, Kamat PV (2016). Intriguing optoelectronic properties of metal halide perovskites. Chem. Rev..

[29] de Weerd C (2018). Efficient carrier multiplication in cspbi3 perovskite nanocrystals. Nat. Commun..

[30] Nie W., Tsai H., Asadpour R., Blancon J.-C., Neukirch A. J., Gupta G., Crochet J. J., Chhowalla M., Tretiak S., Alam M. A., Wang H.-L., Mohite A. D. (2015). High-efficiency solution-processed perovskite solar cells with millimeter-scale grains. Science.

[31] Yang W. S., Noh J. H., Jeon N. J., Kim Y. C., Ryu S., Seo J., Seok S. I. (2015). High-performance photovoltaic perovskite layers fabricated through intramolecular exchange. Science.

[32] Shi D., Adinolfi V., Comin R., Yuan M., Alarousu E., Buin A., Chen Y., Hoogland S., Rothenberger A., Katsiev K., Losovyj Y., Zhang X., Dowben P. A., Mohammed O. F., Sargent E. H., Bakr O. M. (2015). Low trap-state density and long carrier diffusion in organolead trihalide perovskite single crystals. Science.

[33] Coulter JE, Manousakis E, Gali A (2013). Limitations of the hybrid functional approach to electronic structure of transition metal oxides. Phys. Rev. B.

[34] Kim S, Kim K, Kang C-J, Min BI (2013). Correlation-assisted phonon softening and the orbital-selective peierls transition in vo2. Phys. Rev. B.

[35] Weber C (2012). Vanadium dioxide: A peierls-mott insulator stable against disorder. Phys. Rev. Lett..

[36] Becker MA (2018). Bright triplet excitons in caesium lead halide perovskites. Nature.

